# Real-time tracking of cell cycle progression during CD8^+^ effector and memory T-cell differentiation

**DOI:** 10.1038/ncomms7301

**Published:** 2015-02-24

**Authors:** Ichiko Kinjyo, Jim Qin, Sioh-Yang Tan, Cameron J. Wellard, Paulus Mrass, William Ritchie, Atsushi Doi, Lois L. Cavanagh, Michio Tomura, Asako Sakaue-Sawano, Osami Kanagawa, Atsushi Miyawaki, Philip D. Hodgkin, Wolfgang Weninger

**Affiliations:** 1 Immune Imaging Program, Centenary Institute for Cancer Medicine and Cell Biology, Newtown, New South Wales 2042, Australia; 2 Division of Immunology, Walter and Eliza Hall Institute of Medical Research, Parkville, Victoria 3052, Australia; 3 Department of Medical Biology, University of Melbourne, Melbourne, Victoria 3052, Australia; 4 Cell Innovator Co., Ltd., Fukuoka 812-8581, Japan; 5 Laboratory for Autoimmune Regulation, RIKEN Research Center for Allergy and Immunology, Yokohama 230-0045, Japan; 6 Laboratory for Cell Function and Dynamics, Brain Science Institute, RIKEN, Saitama 351-0198, Japan; 7 Discipline of Dermatology, Sydney Medical School, University of Sydney, Sydney, New South Wales 2006, Australia; 8 Department of Dermatology, Royal Prince Alfred Hospital, Camperdown, New South Wales 2050, Australia

## Abstract

The precise pathways of memory T-cell differentiation are incompletely understood. Here we exploit transgenic mice expressing fluorescent cell cycle indicators to longitudinally track the division dynamics of individual CD8^+^ T cells. During influenza virus infection *in vivo*, naive T cells enter a CD62L^intermediate^ state of fast proliferation, which continues for at least nine generations. At the peak of the anti-viral immune response, a subpopulation of these cells markedly reduces their cycling speed and acquires a CD62L^hi^ central memory cell phenotype. Construction of T-cell family division trees *in vitro* reveals two patterns of proliferation dynamics. While cells initially divide rapidly with moderate stochastic variations of cycling times after each generation, a slow-cycling subpopulation displaying a CD62L^hi^ memory phenotype appears after eight divisions. Phenotype and cell cycle duration are inherited by the progeny of slow cyclers. We propose that memory precursors cell-intrinsically modulate their proliferative activity to diversify differentiation pathways.

## Abstract (web summary)

CD8^+^ memory T cells appear during infection via a process of selection and differentiation that remains poorly understood. Using a fluorescent indicator of cell cycle progression, Kinjyo *et al*. show that slow-cycling memory precursors are derived from fast-cycling-activated T cells in influenza-infected mice.

## Graphical abstract

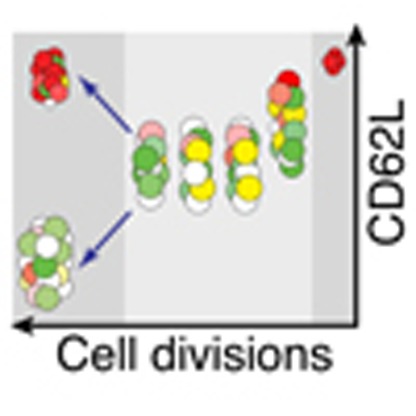

CD8^+^ T cells are crucial for the fight against intracellular pathogens and tumorigenic cells through their capacity of targeted cytolysis. After encounter with an antigen, naive T cells initiate proliferation and differentiate into effector cells equipped with cytotoxic molecules and cytokines. Following eradication of foreign or tumour antigens, the effector population contracts and leaves behind a smaller pool of antigen-specific memory T cells that achieve quick recall responses upon antigen re-encounter^1^,^2^,^3^,^4^.

Although the generation of CD8^+^ memory T cells is a defining feature of adaptive immune responses, how exactly memory T cells develop during primary immune responses has remained a controversial subject. Proposed models include a conventional linear differentiation pathway, whereby naive T cells go through consecutive effector, effector memory (Tem) and then central memory (Tcm) stages^5^,^6^, as well as the decreasing potential and progressive differentiation model where the duration and strength of activating signals regulate the differentiation of memory cells^7^,^8^. Alternatively, lineage fate may already be determined during the first division of naive T cells giving rise to progeny with different fates (asymmetric division model)^9^,^10^. Recent reports that utilized barcoding or congenic marking of individual T cells have proposed that heterogeneous T-cell families with divergent expansion histories and cell fates arise during primary immune responses^11^,^12^. The existence of these partially conflicting models indicates that we require a better understanding of memory T-cell generation.

In all of these models, cell division plays a key role not only by regulating available T-cell numbers^13^,^14^ but also potentially by contributing to the diversification of differentiation pathways. Following the initial encounter of cognate antigen, quiescent naive CD8^+^ T cells initiate proliferation supported by interleukin (IL)-2 (refs 15, 16). The regulation of cell cycle activity is critical for the clonal expansion of effector cells and secondary response of memory cells^17^,^18^,^19^, and is also potentially involved in the stepwise differentiation into memory T cells^20^,^21^,^22^,^23^ (division-linked differentiation). Given the importance of cell cycle progression in immune responses, T-cell proliferation has been analysed extensively. Bromodeoxyuridine (BrdU) and cell cycle marker staining have been used to examine turnover rate or identify proliferating populations but cannot be applied for analysis of real-time cell cycle progression. Most of the efforts have focused on dissecting proliferation dynamics at the population level using cell trace dyes^24^,^25^. These approaches are limited to examining proliferation history until the time when the dye is diluted out. Thus, during critical phases of adaptive immunity, in particular, at a time when memory T-cell precursors first appear *in vivo*, very little information is available on the proliferative signature of T cells. In addition, dynamic studies that determine the correlation between proliferative behaviour and cell fate plasticity of individual T cells within populations over prolonged time periods have not been performed.

To gain insight into these outstanding questions, we utilized transgenic mice that express the fluorescent ubiquitination-based cell cycle indicator (‘Fucci’), in which cells become reversibly fluorescent depending on their cell cycle state^26^. This enabled us to investigate the dynamics of cell cycle progression at the single-cell level during immune responses *in vivo* and *in vitro*. Correlation of the differentiation state of T cells and proliferative activity revealed that naive T cells initially undergo vigorous proliferation after the encounter of antigens. After nine or more division cycles, a subpopulation of T cells separated from the fast cyclers characterized by slowing down of proliferation speed and paralleled by acquisition of a central memory precursor phenotype. Slow cycling and phenotype were an inherited feature of these cells. Thus, our findings suggest that activated T cells can reset their cell cycle machinery to initiate memory cell differentiation programmes separately from the fast-cycling effector pool.

## Results

### Fucci mice facilitate tracking of the cell cycle in T cells

**Figure 1:**
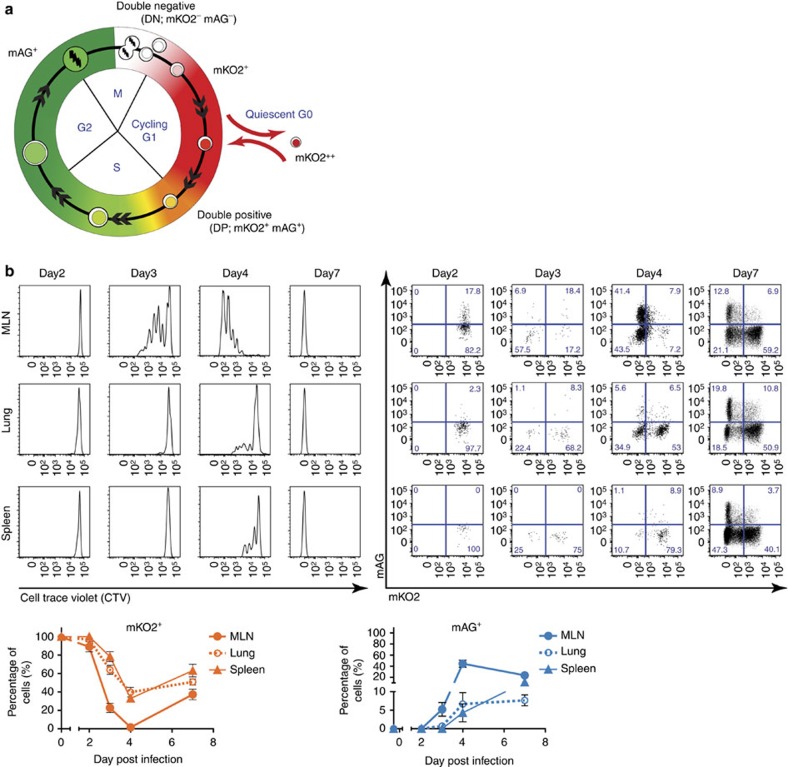
Dynamic cell cycle progression of virus-specific CD8^+^ T cells shown by Fucci probes. (**a**) Fucci fluorescent signals in cell cycle dynamics. The originally designed Fucci system, in which mutually expressed Fucci probes correspond to each cell cycle phase. The cells in G_0/1_ show high intensity of mKO2-hCdt1(30/120), and S/G_2_/M cells represent the accumulation of mAG-hGem(1/110). (**b**) Magnetic-activated cell sorting-purified Fucci/OT-I (CD45.2^+^) cells were labelled with Cell Trace Violet (CTV) dye and transferred into CD45.1^+^ recipients prior to intranasal (i.n.) PR8-OVA influenza A virus infection. Representative flow plots show the CTV dye dilution profile and mAG versus mKO2 expression level. Bottom panels depict the percentage of mKO2^+^ (orange lines) or mAG^+^ (blue lines) from all mice (*n*=9). Data are presented as means±s.e.m.

In Fucci mice, cells in G_0_/G_1_ and S/G_2_/M cell cycle phases express mKusabira-Orange 2 (mKO2) and mAzami-Green (mAG), respectively^26^ (Fig. 1a). The schematic in Fig. 1a represents the originally designed Fucci probe pattern for cell cycle progression, where Fucci probes are matched to cell cycle phases^26^. To dissect proliferative kinetics of CD8^+^ T cells after antigen activation, Fucci (Fucci-G_1_/Fucci-SG_2_M-double transgenic) mice were crossed with T-cell receptor transgenic OT-I mice^27^, in which CD8^+^ T cells recognize OVA_257–64_ (SIINFEKL) peptide presented in the context of the major histocompatibility complex-I. As expected from the previous observation that Fucci cells in quiescent G_0_ phase were mKO2^++^ (ref. 28), naive CD8^+^ T cells isolated from naive Fucci/OT-1 mice were predominantly mKO2^++^mAG^−^ (mKO2^++^; Supplementary Fig. 1a), reflecting their resting state. Following stimulation with SIINFEKL peptide *in vitro*, we found that mKO2^++^ naive cells became mKO2^+^mAG^+^ (double positive), mKO2^−^ mAG^+^ (mAG^+^) or mKO2^−^mAG^−^ (double negative (DN)) after entry into the first cell cycle (Supplementary Fig. 1a; 20 h). Cell cycle profiling using DNA stains confirmed that mAG^+^ cells exhibited higher DNA content than 2 N corresponding to S/G_2_/M phases, and mKO2 positivity was matched to the 2 N state (Supplementary Fig. 1b). At time points when CD8^+^ T cells are known to proliferate vigorously, cycling cells were found as mAG^+^ or DN cells with very low mAG or mKO2 intensity (Supplementary Fig. 1a; 50 h). The DN state arises from the gap between the degradation of mAG-hGeminin(1/110) and accumulation of mKO2-hCdt1(30/120) at the early G_1_ phase^26^. Thus, when cells cycle with a short G_1_ phase, a substantial fraction of cells may be found as DN (Supplementary Note 1). In addition, we cannot formally exclude a certain degree of heterogeneity in Fucci transgene expression, which could also contribute to the DN state in the current transgenic mice. Therefore, in this study we decided to take advantage of the Fucci system by mainly focusing on mAG- or mKO2-positive cells to track the real-time proliferative dynamics *in vivo* during an immune response, which could not be addressed with classical methods.

### Slow-cycling memory precursors appear in influenza infection

To examine the cell cycle kinetics of individual virus-specific CD8^+^ T cells in the course of infection, we adoptively transferred Fucci/OT-I cells to recipient mice that were subsequently infected with influenza A virus PR8 engineered to express ovalbumin (PR8-OVA)^29^. As early as 2 days post infection (p.i.), Fucci/OT-I cells in the mediastinal lymph nodes (MLN) entered the cell cycle as indicated by the transition from mKO2^++^ to the double-positive state, before the first dilution of the cell trace dye became detectable (Fig. 1b). By day 4 p.i., the percentage of mKO2^+^ cells dropped in MLNs, lungs and spleens while mAG^+^ cells increased (Fig. 1b, right). At the peak of infection (day 7 p.i.), when cells had diluted out the cell trace dye after nine or more divisions, the presence of a high percentage of mAG^+^ indicated the continuance of intensive proliferation (Fig. 1b). Lung sections on day 7 p.i. confirmed the presence of mAG^+^ cells *in situ*, indicating that virus-specific CD8^+^ T cells continued to divide within the effector site^30^ (Supplementary Fig. 2). Importantly, at the memory phase (day 32 p.i.), most of Fucci/OT-I cells displayed a CD44^hi^KLRG-1^lo^IL-7Rα^hi^ memory phenotype and became mKO2^++^mAG^−^, indicating that they became quiescent (Supplementary Fig. 3).

**Figure 2:**
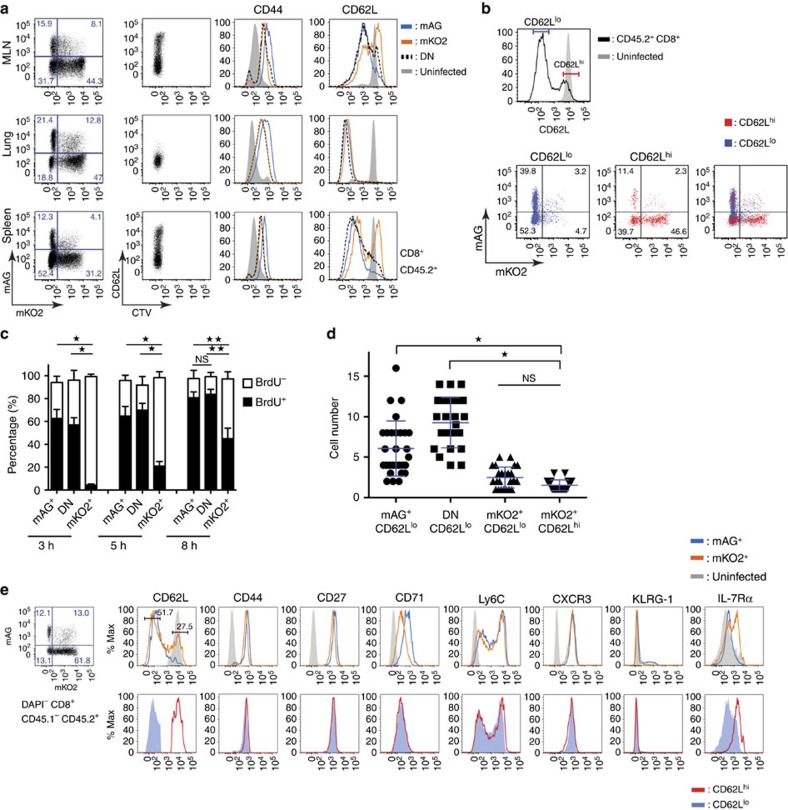
The mKO2-positive cells reappearing on day 7 p.i. are slower cycling cells. Magnetic-activated cell sorting-purified Fucci/OT-I (CD45.2^+^) cells were labelled with CTV dye and transferred into CD45.1^+^ recipients prior to intranasal (i.n.) PR8-OVA influenza A virus infection. (**a**) Representative flow plots of mediastinal lymph nodes (MLNs), lungs and spleens on day 7 p.i. The histograms show CD44 and CD62L levels for mAG^+^ (blue), mKO2^+^ (orange) and DN (black dot) cells from infected mice against naive cells in uninfected mice (grey solid). Data are representative of three independent experiments with three to five mice each. (**b**) Backgating analysis for expression profiles of mAG versus mKO2 in CD62L^hi^ and ^lo^ subsets from the spleens of mice on day 7 p.i. CD62L^hi^ (red) and ^lo^ (blue) gates were determined as shown in the histogram and compared for mAG versus mKO2 in dot plots. Data are representative of three independent experiments with three to five mice each. (**c**) BrdU incorporation in virus-specific CD8^+^ T cells on day 7 p.i. The percentages of BrdU^+^ cells in mAG^+^, mKO2^+^ or DN subpopulations in spleens were assessed at 3, 5 and 8 h after BrdU intraperitoneal administration on day 7 p.i. Data are representative of three independent experiments with four mice each. Bars show mean with s.d. (*n*=12, *P<0.001, **P<0.01; two-way analysis of variance (ANOVA)). (**d**) mAG^+^CD62L^lo^, DN CD62L^lo^, mKO2^+^CD62L^lo^ and mKO2^+^CD62L^hi^ subsets within the CD8^+^ CD45.2^+^ cell population were sorted on day 7 p.i. as single cells and cultured in rIL-2 (10 ng ml^−1^) containing medium. The wells with a single cell at the time 0 were observed by time-lapse imaging to count the cell number at 48 h. Data were summarized from three independent experiments. Bars show mean with s.d. (*n*=35, *P<0.001 versus KO^+^CD62L^hi^; NS, not significant. ANOVA). (**e**) Memory-specific cell surface markers on virus-specific CD8^+^ T cells on day 7 p.i. Top panel: histograms of each marker are shown for mAG^+^ (blue line) versus mKO2^+^ (orange line) compared with naive cells in uninfected mice (grey solid). Bottom panel: histograms of each marker are shown for CD62L^hi^ (red line) versus CD62L^lo^ (blue solid). Data are representative of three independent experiments with three mice each.

Taking advantage of the Fucci system, we dissected the cell cycle status of highly divided cells after complete dye dilution. Consistent with previous reports^31^, a population of CD44^hi^CD62L^hi^ Tcm-like cells appeared on day 7 p.i. in the spleens and MLNs, but not in the lungs (Fig. 2a). When gating on CD62L^hi^ cells, approximately half of them were mKO2^+^mAG^−^, indicating slowing of their cell cycle as compared with CD62L^lo^ effector T cells, which remained mAG^+^ (Fig. 2b). Conversely, mKO2^+^ cells in the MLN and spleen contained a sizeable population of CD62L^hi^ cells, while mAG^+^ cells were largely CD62L^lo^ (Fig. 2a).

To confirm that the mKO2^+^ T cells that reappeared on day 7 p.i. were indeed slower cyclers than their mAG^+^ counterparts, mice received a single intraperitoneal injection of BrdU on that day (Fig. 2c). After 3 h, while about 60% of mAG^+^ cells incorporated BrdU, mKO2^+^ cells mostly stayed BrdU^−^. The proportion of BrdU^+^mKO2^+^ cells increased over the next 5 h, but never reached the level of mAG^+^ cells. As only cells in the S phase can incorporate BrdU, BrdU^+^mKO2^+^ cells must be the cells that have newly entered the G_1_ phase after recent cytokinesis. The BrdU^−^mKO2^+^ cells remaining after 8 h likely contained not only the cells that have just moved from the G_2_ to M phase but also those that stayed in the G_1_ phase for 8 h, indicating that the mKO2^+^ population contained the slower cycling cells.

Furthermore, culture of sorted single cells isolated on day 7 after PR8-OVA infection showed significantly less expansion of mKO2^+^ cells compared with mAG^+^CD62L^lo^ cells *ex vivo* (Fig. 2d). Thus, activated virus-specific Fucci transgenic CD8^+^ T cells were found as mAG^+^ or DN cells during the initial vigorous expansion phase, and some of them slowed down their cell cycle speed paralleled by the accumulation of mKO2-hCdt1(30/120) on day 7 p.i.

Cell surface marker profiling revealed that both mAG^+^ and mKO2^+^ cells on day 7 p.i. displayed an activated phenotype with elevated expression of CD44, CD27, Ly6C and CXCR3 (Fig. 2e). The mAG^+^ population showed higher expression of CD71 (transferrin receptor protein 1), indicating their highly proliferative status^32^,^33^, and slightly higher expression of KLRG-1. Consistent with a memory precursor phenotype, mKO2^+^ cells expressed higher levels of IL-7Rα. In addition, messenger RNA expression levels of transcription factors ELF4 and KLF2, which are known to bind and activate CD62L and S1PR1 (sphingosine-1-phosphate receptor) promoters^34^,^35^,^36^, were upregulated in mKO2^+^ cells similarly to CD8^+^ memory control (Supplementary Fig. 4). In contrast, the expression levels of interferon (IFN)-γ and IL-2 were higher in mAG^+^ cells than mKO2^+^ cells (Supplementary Fig. 4). These data suggest that slow-cycling mKO2^+^ cells exhibit a Tcm precursor phenotype.

### Memory precursors arise from highly proliferative T cells

**Figure 3:**
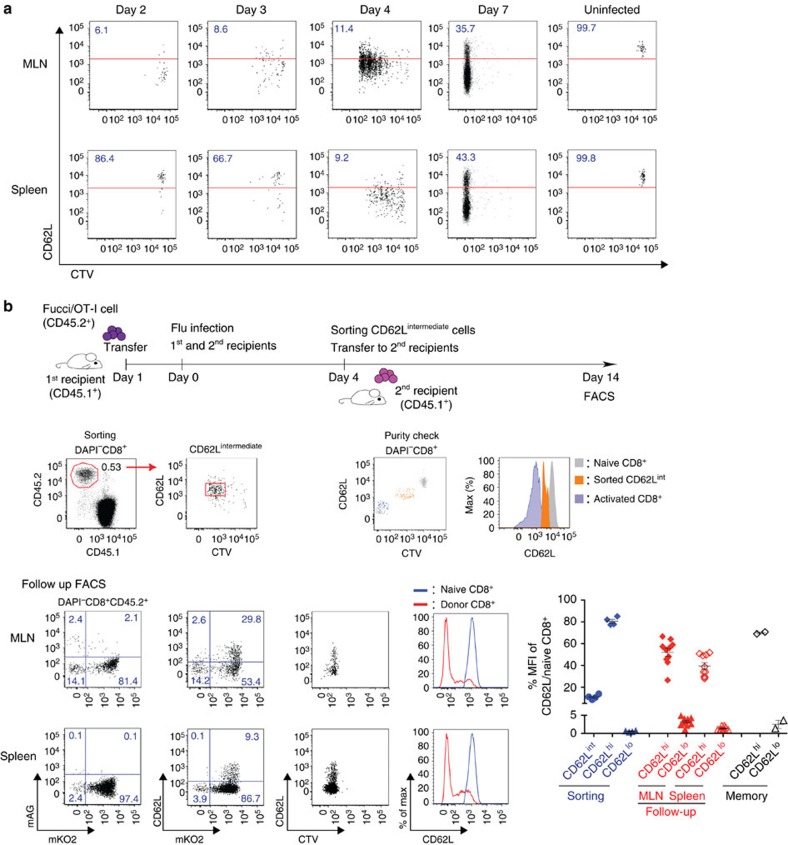
Proliferating virus-specific CD8^+^CD62L^int^ cells segregate into CD62L^hi^ and CD62L^lo^ subsets. Magnetic-activated cell sorting-purified Fucci/OT-I (CD45.2^+^) cells were labelled with CTV dye and transferred to CD45.1^+^ recipients prior to PR8-OVA influenza A virus infection. (**a**) Surface expression of CD62L against CTV dilution on virus-specific CD8^+^ T cells in MLNs and spleens on days 2, 3, 4 and 7 p.i. Data are representative of three independent experiments with three to five mice each. (**b**) CD62L^int^ Fucci/OT-I cells in the 4th–8th divisions were sorted from splenocytes on day 4 p.i. and transferred into the concurrently infected 2nd recipients. Ten days after transfer, 2nd recipients were analysed for the change of CD62L level. The percentage of CD62L mean fluorescence intensities against naive CD8^+^ T cells is shown to compensate differences in fluorescence between sorter and analyzer instruments. Symbols show mean with s.d. (*n*=7). Data are representative of three independent experiments with three mice each.

The fact that mKO2^+^CD62L^hi^ cells had diluted cell tracker dye indicated that they may have arisen from the effector cell pool. Alternatively, they may have developed as an independent population with a constantly high CD62L level that initially went through a similar expansion as effector T cells. Following activation, naive T cells proliferated and also downregulated CD62L expression^37^ (Fig. 3a). By day 4 p.i., most of the virus-activated cells showed intermediate level of CD62L, as a relatively homogenous population, compared with the distinct CD62L^hi^ and CD62L^lo^ subsets present on day 7 p.i. To determine whether both CD62L^hi^ and CD62L^lo^ populations developed from the same activated CD62L^int^ T-cell pool, we sorted CD44^hi^CD62L^int^ T cells within the 4–8th division on day 4 p.i., and transferred them to secondary recipient mice that were concurrently infected with PR8-OVA. After 10 days, we found that the transferred T cells continued to proliferate, and that they gave rise to both CD62L^lo^ and CD62L^hi^ populations (Fig. 3b). Therefore, virus-activated T cells retain their potency to become effector or memory cells during the early expansion phase, and at some later time point, split into either effector or memory differentiation pathways after multiple division cycles.

### Real-time tracking of CD8^+^ T-cell divisions *in vitro*

**Figure 4:**
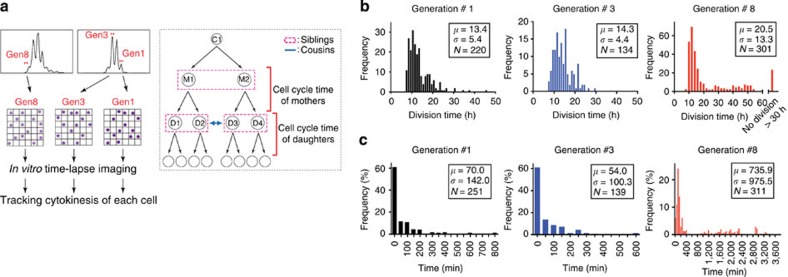
Real-time tracking of Fucci CD8^+^ T-cell divisions at the single-cell level. (**a**) Left: scheme about tracking cytokinesis of individual cells. Magnetic-activated cell sorting-purified Fucci CD8^+^ T cells were labelled with CTV dye and stimulated with plate-bound anti-CD3ε (1.0 μg ml^−1^) and anti-CD28 mAb (0.5 μg ml^−1^). Cells in the 1st, 3rd and 8th generation were sorted and placed into microgrids. Time-lapse imaging was performed every 3–4 min for 66–90 h and sequential cytokinesis was traced to measure cell cycle time. Right: scheme of the family tree of tracked cells. First, single cells found as only one cell in microgrid wells were tracked for several divisions. Cells from the 1st division are designated ‘mothers (M1 and M2)’ and the cells from the 2nd divisions ‘daughters (D1, D2 or D3, D4)’. In addition, ‘daughter’ groups are distinguished as ‘siblings (D1 versus D2 or D3 versus D4)’ from the same mother or ‘cousins (D1/D2 versus D3/D4)’ from different mothers. (**b**) The distribution of measured cell cycle times are shown in each histogram. Each histogram represents sorted cells in the 1st (black), 3rd (blue) and 8th (red) generation. Data were summarized from three independent experiments. Mean (*μ*), s.d. (*σ*) and number of events (*N*) are shown. (**c**) The percentage of frequency for mKO2^+^ phase duration of cells from the 1st, 3rd and 8th generations is shown in the histograms. Each histogram represents sorted cells in the 1st (black), 3rd (blue) and 8th (red) generations. Data were summarized from three independent experiments. Mean (*μ*), s.d. (*σ*) and number of events (*N*) are shown.

Recent evidence suggests that during primary immune responses, individual naive T cells give rise to separate families with distinct differentiation profiles, namely highly proliferative effector and less-expanding Tcm families^11^,^12^,^38^. These observations are somewhat at odds with our data that suggest that Tcm precursors undergo initial vigorous proliferation prior to switching to a slow-cycling mode. If indeed distinct proliferative families would be generated, one would expect to find heterogeneity in cell cycle times, and a high correlation (inheritance) of times between generations within individual T-cell families. To better understand the cycling characteristics of T-cell families, we measured actual cell cycle duration by time-lapse imaging *in vitro*. We sorted *in vitro* activated Fucci CD8^+^ T cells from early- (first and third) and late- (eighth) division generations, and performed single-cell time-lapse imaging of cells placed in a microgrid array for up to 90 h (Fig. 4a; Supplementary Movie 1). Next, we manually tracked each cytokinesis by identifying individual cells, and measured their cell cycle duration. Since it was not possible to determine the exact time to the first cytokinesis in the acquired movies, we started measurements after the observed first cytokinesis for two subsequent division rounds (Fig. 4a; Supplementary Fig. 5). To distinguish each cell arising from the same progenitor, we named the cells from the 1st divisions as ‘mothers (M1 and M2)’ and the cells from the 2nd divisions as ‘daughters (D1, D2 or D3, D4)’. For further analysis, daughters’ groups are distinguished as ‘siblings (D1 versus D2, D3 versus D4)’ from the same mother or ‘cousins (D1/D2 versus D3/D4)’ originating from different mothers from the same initial progenitor (C1). By collecting cell cycle time data, we found cells from the 1st and 3rd generations showed similar and fast cell cycle times with a mean of 13.4±5.4 and 14.3±4.4 h, respectively (Fig. 4b). In generation-8 cells, in addition to fast-cycling cells (early dividers) with similar proliferation and inheritance features to the cells from earlier generations, we also observed the occurrence of a subset of slow- and non-cycling cells, some of which had division times of more than 24 h (Fig. 4b; Supplementary Fig. 6a). This was evidenced by the fact that their first cytokinesis was not observed until the 2nd imaging day (late dividers) and their daughters did not divide again before the end of the observation period (Fig. 4b, data below). Consistent with their long division times, the slow-dividing cells had markedly increased periods of mKO2 positivity (Fig. 4c; Supplementary Fig. 6b). These differences in division patterns between the 1st/3rd generation and 8th generation were highly significant (Supplementary Fig. 6c).

**Figure 5:**
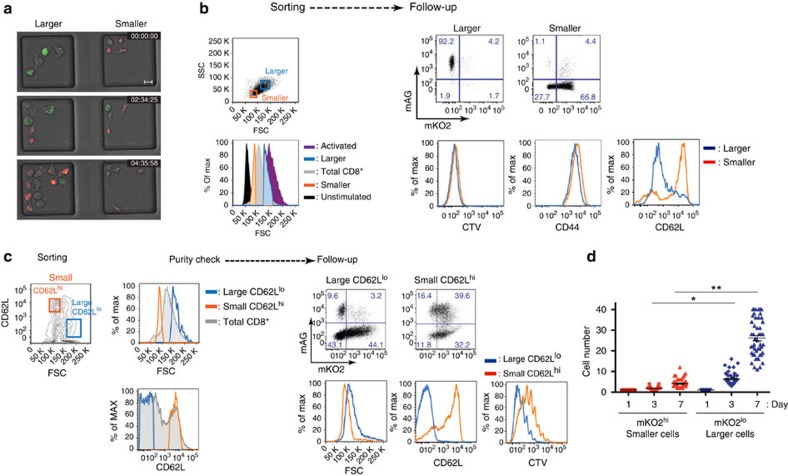
Cell size correlated with different CD62L expression levels and proliferative activity. (**a**) Representative snapshots from a time-lapse movie of sorted 8th-generation cells between the 3rd and 4th imaging day. The left well with larger sized cells and the right well with smaller sized cells were imaged on the same microgrid array simultaneously under the same condition. The snapshots from a representative movie (Supplementary Movie 2) from three independent experiments are shown (scale bar, 10.0 μm). (**b**) Flow cytometric analysis of CD62L expression and cell size of *in vitro* stimulated CD8^+^ T cells (plate-bound anti-CD3ε (1.0 μg ml^−1^) and anti-CD28 mAb (0.5 μg ml^−1^)) on day 7. Left: sorting gate for smaller or larger sized cells. Right: plots of the mAG versus mKO2 levels in smaller and larger sized cells after 3 days of culture with 10 ng ml^−1^ of rIL-2. The histograms of each marker are shown for smaller sized cells (orange line) versus large cells (blue line). Data are representative of three independent experiments. (**c**) The larger CD62L^lo^ and smaller CD62L^hi^ donor Fucci/OT-I cells sorted from splenocytes of PR8-OVA-infected mice on day 7 p.i. were cultured to compare proliferative activity. Left: sorting gate for larger CD62L^lo^ (blue line) and smaller CD62L^hi^ (orange line) cells. Sorted samples were checked for purity. After labelling with CTV dye, cells were cultured with 10 ng ml^−1^ of rIL-2 for 3 days. Right top: dot plots depicting the level of mAG versus mKO2 expression between the larger CD62L^lo^ cells and the smaller CD62L^hi^ cells after following culture. Right bottom: histograms of CD62L level and CTV dye dilution are shown for the larger CD62L^lo^ (blue line) and smaller CD62L^hi^ (orange line) cells. Data are representative of three independent experiments with three mice each. (**d**) Cell numbers from sequential divisions of cells sorted based on cell size. The smaller sized mKO2^hi^ and larger sized mKO2^lo^ donor Fucci/OT-I cells from splenocytes of PR8-OVA-infected mice on day 7 p.i. were plated by single-cell sorting. The sorted cells were cultured in rIL-2 (10 ng ml^−1^) containing medium. Wells with a single cell at time 0 were followed up by imaging on days 1, 3 and 7, and the number of cells in each well was counted. *P<0.05, **P<0.001 by the Mann–Whitney’s *U*-test.

Furthermore, we found that the cells with longer mKO2^+^ phases were smaller in cell size than fast-proliferating mAG^+^ cells, and were mostly CD62L^hi^ (Fig. 5a,b; Supplementary Movie 2). A similar small CD62L^hi^ cell population was identified during influenza virus infection *in vivo* (Fig. 5c). When we cultured sorted small mKO2^+^CD62L^hi^ and large mKO2^+^CD62L^lo^ cells *ex vivo*, the larger cells were more proliferative and stayed CD62L^lo^, while the smaller cells stayed CD62L^hi^ and proliferated less (Fig. 5c,d).

### Correlation of cell cycle times between T-cell relatives

**Figure 6:**
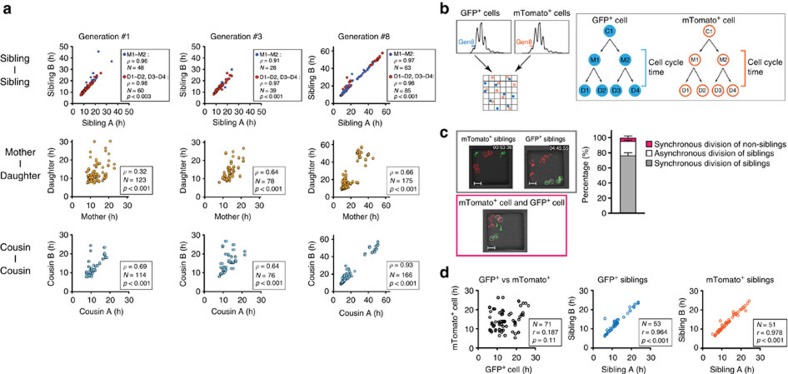
Cell cycle time regulation is shared by progenies in kinship. (**a**) Correlation analysis of cell cycle times between ‘siblings’ (top), ‘mother–daughter’ (middle) and ‘cousins’ (bottom) from sorted 1st, 3rd and 8th generations. Data are cumulative of three independent experiments from Fig. 4. Significance of correlation was determined by Spearman’s rank correlation coefficient (*ρ*), significance (*P*) and number of events (*N*). (**b**) Scheme shows the strategy for sorting and tracking of non-relative GFP^+^ or membrane-tdTomato^+^ (mTomato^+^) cells from their respective 8th generations. (**c**) Left: representative snapshots with synchronous divisions of two mTomato^+^ or two GFP^+^ cells (top) or with a mTomato^+^ and a GFP^+^ cell (bottom) (scale bar, 10.0 μm). Right: frequency of observed division types. Data are cumulative from three independent experiments. Bars represent mean±s.d. (*N*=45). (**d**) Correlation analysis of cell cycle times between GFP^+^ and mTomato^+^ cells. Data are cumulative from three independent experiments. Significance of correlation was determined by Spearman’s rank correlation coefficient (*ρ*), significance (*P*) and number of events (*N*).

To examine whether there was evidence for inheritance of cell cycle times within division trees arising from individual T cells, we analysed the correlation of horizontally (that is, siblings and cousins) and vertically (that is, mother versus daughter) related cell cycle times (Figs 4a and 6a). In all generations, siblings showed a very strong correlation, while mothers and daughters exhibited a weaker correlation of division times. The correlation between cousins was higher than that of mothers and daughters. Nevertheless, cell cycle times were not strictly identical between generations, but rather showed variations with s.d. of more than 4 h.

The fact that siblings showed high correlation of cycling times could be cell intrinsic, due to equal partitioning of the cycling machinery in the two daughters after cytokinesis, or due to environmental factors within the microgrids. To distinguish between these possibilities, we performed time-lapse imaging of separately stimulated CD8^+^ T-cell populations expressing either cytoplasmic green fluorescent protein (GFP)^39^ or membrane-targeted tdTomato^40^ seeded in the same wells (Fig. 6b). Analysis showed that same-colour siblings divided synchronously (Fig. 6c), but non-relatives from differentially-coloured progenitors divided without correlation (Fig. 6d), indicating that cell intrinsic, rather than environmental, factors determine cycling times of activated T cells.

### Inheritance of cell cycle times in sequential divisions

**Figure 7:**
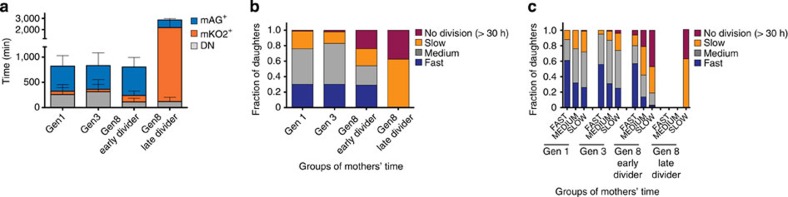
Inheritance of cell cycle time during late divisions. (**a**) The duration of mAG^+^, mKO2^+^, DN phases was measured from time-lapse imaging of each generation. Data are summarized from three independent experiments. The bar graphs represent means with s.d. Cells from the 8th generation were further grouped into ‘early divider’ (divided on the 1st day of imaging) and ‘later divider’ (divided on the 2nd day of imaging). Data were summarized from three independent experiments from Fig. 4. (**b**) Fractions of daughters grouped according to their cell cycle time (fast<600 min, 600 min<medium<800 min, slow>1,000 min and no division >30 h until the end of imaging) are shown. Each bar represents the cells from the sorted 1st, 3rd or 8th generation. Data were summarized from three independent experiments from Fig. 4. (**c**) Inheritance of cell cycle time from mothers to daughters. Fractions of daughters derived from the mothers grouped according to cell cycle time (fast<600 min, 600 min<medium<800 min, slow >1,000 min and no division >30 h until the end of imaging) are shown. Data are summarized from three independent experiments from Fig. 4. Significance value for the ‘slow’ and ‘no division’ fractions of each generation were calculated using the *χ*^2^-test (*P*<0.001).

Thus far, our data have shown that within a cycling population of activated T cells two basic cycling patterns with respective early and late dividers can be identified in the 8th generation (Fig. 7a). To determine whether these patterns were an inherited feature, we arbitrarily categorized cycling times of *in vitro* stimulated Fucci CD8^+^ T cells into fast (<600 min), medium (600–800 min), slow (>1,000 min) and undivided cells. This analysis revealed that 8th-generation cells gave rise to a much higher proportion of slow dividers and undivided daughters as compared with earlier generations (Fig. 7b). Inheritance was then tested by grouping mothers into fast, medium and slow dividers, and comparing them with the division category of daughters (Fig. 7c). In the 1st and 3rd generation, both fast- and slow-cycling mothers gave rise to a similar distribution of cycling times of progeny, which is consistent with stochastic resetting of cycling times between generations as described above. In contrast, in the 8th-generation slow-dividing mothers gave rise to a much higher proportion of slow- or non-dividing daughters than faster dividing mothers (Fig. 7c). We concluded that at this stage slow division was an intrinsic, inherited feature of Tcm-like cells arising *in vitro*.

### Transcriptome analysis of small slow-cycling CD8^+^ T cells

**Figure 8:**
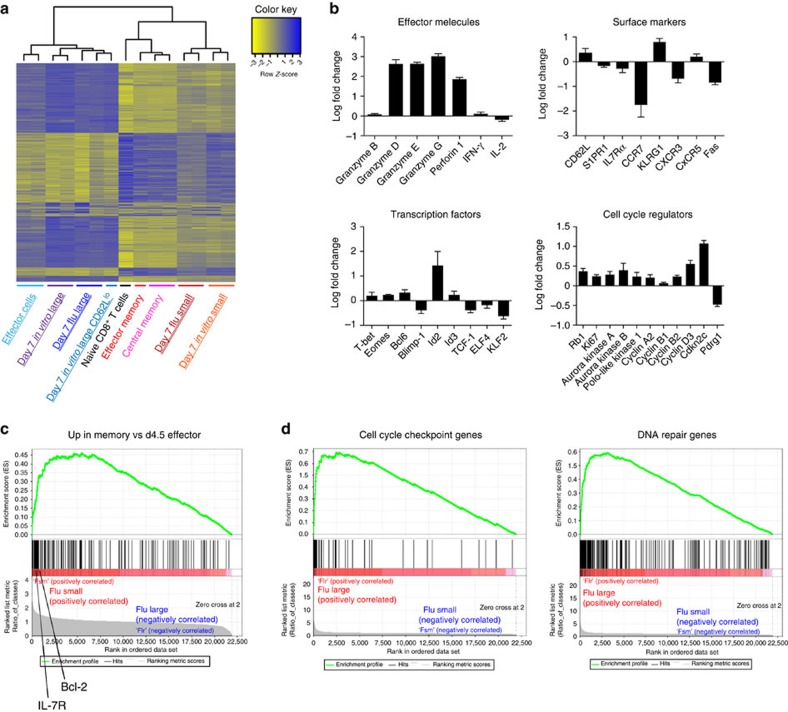
Microarray analysis of larger and smaller sized T cells from influenza-infected mice. RNA samples were prepared from sorted populations of larger or smaller sized cells from spleens of influenza virus PR8-OVA-infected mice on day 7 p.i. or from *in vitro* 7 days culture after stimulation with plate-bound anti-CD3ε (1.0 μg ml^−1^) and anti-CD28 mAb (0.5 μg ml^−1^). Effector T-cell control samples were prepared from SIINFEKL (100 ng ml^−1^) stimulated OT-I cells after 4 days of *in vitro* culture and sorted as CD8^+^CD44^hi^CD62L^lo^. Control *bona fide* effector memory and central memory T cells were sorted from the spleens of PR8-OVA-infected mice on day 42 p.i. Naive cells were sorted as CD8^+^CD44^lo^CD62L^hi^ cells from uninfected C57BL/6 mice. Duplicate samples were prepared from independent experiments. (**a**) Clustering analysis and heatmap of gene expression values to depict the similarity of gene profiles between samples for the 934 significant genes (*P*<0.01). The colour key shown on the top illustrates the relative expression level across all samples: blue represents expression above the mean and yellow represents expression lower than the mean. The *in vivo* samples of interest are labelled with a double underline and *in vitro* samples are labelled with a single underline. (**b**) The effector or memory phenotype-associated genes were compared between duplicate samples of larger and smaller sized cells sorted from the spleens of infected mice on day 7 p.i. The log fold change of the expression value (the larger cells/the smaller cells) is shown as black bars. The means with s.d. are shown. (**c**) Gene set enrichment analysis (GSEA) plot shows that the duplicated samples of sorted smaller cells from spleens on day 7 p.i. are enriched for previously reported gene sets for memory CD8^+^ cells (*P*<0.001, false discovery rate (FDR)<0.25). (**d**) GSEA plots showing the enrichment of cell cycle checkpoint or DNA repair gene sets in the duplicate samples of sorted larger cells from spleens on day 7 p.i. (*P*<0.001, FDR<0.25).

To gain further insight into the characteristics of slow-cycling smaller cells that segregated from the activated T-cell pool, we performed a genome-wide microarray expression analysis. Larger and smaller cell-sized CD8^+^ OT-I cells were sorted on day 7 after *in vitro* stimulation or influenza virus infection *in vivo*. In heatmap and clustering analyses, the large cells clustered with effector T-cell control samples, while the smaller cells clustered with memory control samples (Fig. 8a), which indicates that large cells have a closer gene expression pattern with effector cells, but smaller cells are more similar to memory cells. Analysis of selected genes showed that the larger sized cells expressed more cytotoxic molecules and cell cycle regulators associated with their effector phenotype and active proliferation (Figs 5c and 8b). In contrast, smaller sized cells had acquired homing receptors such as CCR7 and CXCR3, and expressed transcription factors such as ELF4 and KLF2, reported to be critical for memory cells (Fig. 8b). Gene set enrichment analysis further demonstrated that smaller sized cells were enriched for genes previously shown to be upregulated in memory cells^41^ (Fig. 8c), suggesting they are on their way to differentiate into memory cells rather than being effector cells. The larger cells were enriched for genes related to the cell division process, DNA replication, cell cycle regulation, microtubule cytoskeleton and the DNA repair process compatible with their proliferative phenotype (Fig. 8d). Taken together, the slower cell cycle times and higher CD62L expression level of small sized T cells, arising during the peak of the influenza response *in vivo* and after eight divisions *in vitro*, match the gene expression profile of *bona fide* memory T cells.

## Discussion

**Figure 9:**
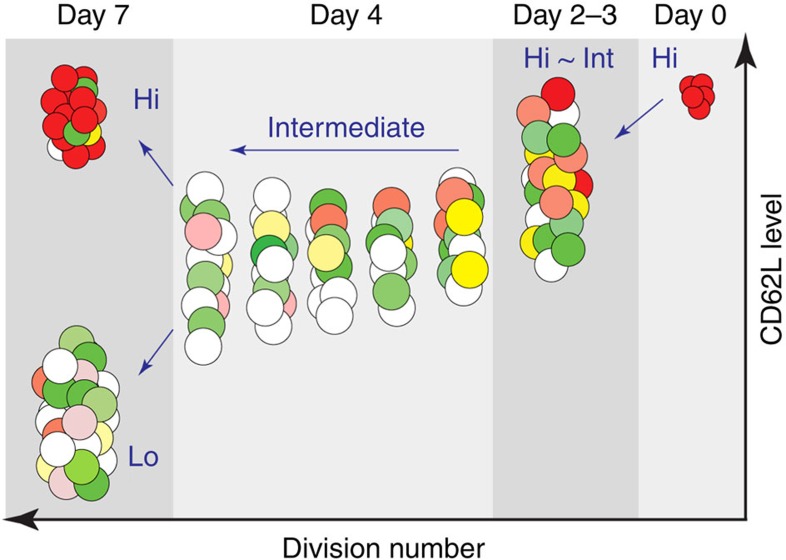
Proposed model for the development of early-memory precursors. Naive Fucci CD8^+^ T cells are mKO2^hi^ small cells in the G_0_ phase with high CD62L expression levels. Following virus infection, antigen-activated CD8^+^ T cells enter the cell cycle and downregulate CD62L expression. During the vigorous expansion phase, most of the activated CD8^+^ T cells increase in size and are found as mAG^+^ or DN cells reflecting fast cell divisions accompanied by intermediate CD62L levels (around days 4–6). On day 7 p.i., the proliferative CD8^+^ T-cell pool starts to segregate into the smaller CD62L^hi^ mKO2^+^ central memory precursor cells, and the larger CD62L^lo^ phenotype cells, which maintain proliferation and differentiation into terminal effector cells.

Proliferation critically determines the quality of immune responses by regulating the number of available effector and memory T cells. In addition, proliferative dynamics are linked to the differentiation state of T cells. Characterization of T-cell cycling dynamics during a time when memory T-cell precursors appear during immune responses has been challenging, due to technical limitations of conventional proliferation assays. Using the Fucci cell cycle reporter system, we have longitudinally dissected the proliferative behaviour of CD8^+^ T cells in the course of immune responses *in vivo* and *in vitro*. We show that memory T-cell precursors initially undergo fast proliferation indistinguishable from effector T cells, and then switch to a heritable slow-cycling mode paralleled by their acquisition of a memory cell phenotype. Our data suggest a memory T-cell differentiation pathway, whereby the fast-cycling T-cell pool maintains a flexible programme that enables direct differentiation into Tcm precursors and effector cells during a phase of anti-viral immune responses when the T-cell population is still expanding (Fig. 9). This plasticity of memory cells seems to be guided by cell intrinsic modulation of cell cycle progression, which potentially protects these cells from exhaustion due to slow-cycling characteristics and small cell size.

The rapid expansion of antigen-specific T-cell clones following encounter of cognate antigen is a cardinal feature of the adaptive immune response, and this process has been examined extensively in a variety of infection and immunization models. While it is well established that cell extrinsic and environmental factors, such as major histocompatibility complex/antigen–T cell receptor (TCR) interactions, co-stimulation and cytokines, as well as specific anatomical locations are critical for the proliferation and differentiation of naive CD8^+^ T cells^42^,^43^,^44^, it is less clear whether lymphocytes have any intrinsic mechanism to regulate their cell cycle duration. Our time-lapse imaging data of cycling T cell *in vitro* demonstrate that T-cell siblings derived from the same mother cell displayed synchronous cell cycle progression during the initial expansion phase (until the 9th generation). Considering that the cell cycle is composed of consecutive G_1_–S–G_2_–M phases, which are regulated by distinct cell cycle checkpoints, it is likely that siblings inherit the founders’ cell cycle machinery components during cytokinesis when T cells clonally expand. Previous reports have revealed that asymmetric division during the first division may act as a mechanism for CD8^+^ T cells fate determination^9^,^10^. Thus, prior to the first division, asymmetric distribution of signalling molecules is established during interactions with antigen-presenting cells, and cells receiving less of such molecules proceed towards central memory cells. While our data do not contradict these results, they suggest that during subsequent divisions, which are more IL-2 dependent and can occur without further antigen stimulation, asymmetry may not play a role, resulting in daughter cells that more closely resemble each other. Nevertheless, division times between unrelated fast-cycling cells do show variations, with cycling times apparently reset after each generation. This is reminiscent of B cells, in which the cycling machinery is inherited in each individual generation, but that some, as yet unknown, stochastic process, randomizes division times equally for the two daughters after each division^45^,^46^.

Previous studies have shown that a single naive CD8^+^ T cell can achieve both effector and memory subset differentiation^47^,^48^, supporting the idea that distinct T-cell subsets may develop by intraclonal diversification during immune responses. Additional evidence using barcoded or congenically labelled cell-transfer methods suggested that naive T cells have the potential to respond with heterogeneity to the initial antigen stimulation, resulting in the generation of diverse families with different capacity for expansion, as well as development into effector or central memory cells^11^,^12^. Nevertheless, it is conceivable that certain T-cell families are heterogeneous and may change their proliferative behaviour over time. Indeed, even in proliferative families, which were considered to be composed of fast-cycling cells of effector phenotype, CD62L^hi^ were present^11^. Whether such intra-familial phenotypic heterogeneity is also reflected by varied cell cycle times would require the longitudinal tracking of individual dividing cells over several generations. In our experiments, we measured the cell cycle times of individual CD8^+^ T cells during sequential cytokinesis using time-lapse imaging and examined the relationship between families or T cells within a given family. Consistent with the mentioned studies^11^,^12^,^49^, T-cell cycle times between progenies from the same founder showed high correlation while they varied between families. Importantly, however, a slower cycling subpopulation of central memory-like cells appeared after many divisions from the fast-cycling T-cell pool, suggesting that cycling capacity is not fixed within T-cell families. Additional support for this hypothesis comes from our adoptive transfer experiments using CD62L^int^CD44^hi^CD8^+^ T cells in the 4–8th division generations, which excluded weakly activated T cells and/or ‘late comers’ to the antigen-presenting site, as those cells are known to preferentially obtain a memory phenotype. Upon transfer these cells maintained the capability to differentiate into both CD62L^hi^ and CD62L^lo^ cells in the presence of cognate antigen. Although our studies do not exclude that small Tcm cell families arise early during immune responses, potentially through less proliferation, we suggest that fully activated, fast-proliferating T cells keep the capacity for direct differentiation into Tcm precursors during a phase of anti-viral immune responses when the T-cell population is still expanding. The fact that these cells are present in high numbers may indicate that they outweigh Tcm precursors differentiating directly from naive T cells. Future studies will investigate the precise fate of these slow-cycling putative memory-like precursors in long-term adoptive transfer experiments.

An important question that remains to be addressed is how the slow- and non-dividing cells segregate from the activated CD8^+^ T-cell pool after multiple rounds of divisions. Since slow- and non-cycling cells were observed *in vitro* under identical conditions with parallel cells maintaining fast cycling speeds, a cell intrinsic process that resets the cell cycle pace appears to be likely. This intrinsic process may be regulable as the number of generations cells undergo before returning to quiescence is reported to vary with strength of TCR stimulation and availability of costimulatory and cytokine signals^50^. Our gene profiling analyses indicate that small slow cyclers are equipped with a *bona fide* memory cell profile, with lower metabolic activity as compared with fast cyclers. The cells with lower expression of nutrient transporters may terminate proliferation and become smaller in size, resembling memory cells^51^,^52^. On the other hand, larger fast cycler cells are enriched in genes involved in the response to DNA damage and oxidative stress. This may indicate that fast cyclers are those cells that experience higher levels of exogenous stress, which ultimately leads to the known propensity of full-fledged effector T cells to undergo apoptosis. Conversely, slowing of the cell cycle and reducing cell size may protect memory precursor T cells from accumulating further cellular toxic stress, which may be beneficial for their long-term persistence. Further studies using genetic approaches or signalling pathway inhibitors will be required to determine the precise mechanism underlying the bifurcation of cycling dynamics.

In summary, we uncovered the dynamics of cell cycle duration with the advantage of Fucci cell cycle report system to track live T cells in the course of the immune response, and identified a sizeable proportion of Tcm precursors that separate from the activated effector pool by slowing down the cell cycle at the peak of the expansion phase. New insight that some progenies of fully activated and extensively proliferated CD8^+^ T cells have the plasticity to enter the central memory differentiation pathway to escape the contraction phase adds new perspectives for memory development in vaccination and immunotherapeutic strategies.

## Methods

### Mice

C57BL/6 (wild type) and B6.SJL/Ptprc^a^ (CD45.1) mice were purchased from the Animal Research Centre (Perth, Australia). mKO2-hCdt1(30/120) (Fucci-G_1_-#639) and mAG-hGem(1/110) (FucciS/G_2_/M-#474) transgenic mice were described in a previous report^26^,^28^,^53^. Fucci-double transgenic mice (#639/#474) were backcrossed onto the C57BL/6 background, and further crossed with OT-I TCR transgenic^27^ (CD45.2) and B6.SJL/Ptprc^a^ (CD45.1) mouse strains. DPE-GFP transgenic mice that express GFP under the control of the murine CD4 promoter^39^, and mT/mG transgenic mice that express membrane-targeted Tomato fluorescent protein under the chicken β-actin promoter^40^ were described previously. All mice were maintained in specific pathogen-free conditions at the Centenary Institute animal facility. All experiments were performed in accordance with protocols approved by the Animal Ethics Committee at the University of Sydney and the Sydney Local Health District Animal Welfare Committee.

### Adoptive transfers and influenza A virus infection

CD8^+^ T cells from spleen and LNs of Fucci/OT-I mice were purified using anti-CD8α-conjugated microbeads (Miltenyi Biotec) and labelled with 5 μM Cell Trace Violet (CTV, Invitrogen) in pre-warmed PBS at 10^6^ ml^−1^. Labelling was performed by incubating in a 37 °C water bath for 20 min and stopped by adding 100% fetal calf serum (FCS) and followed by incubation in RPMI with 10% FCS. Cells were washed twice using PBS and counted. Labelled Fucci/OT-I cells (10^6^) were adoptively transferred into B6.SJL/Ptprc^a^ or Fucci/B6.SJL/Ptprc^a^ mice. On the next day, the mice were anaesthetized by intraperitoneal injection of Ketamine/Xylazine (80/10 mg kg^−1^) and infected intranasally with 100 plaque-forming units of OVA_257–264_ peptide expressing influenza virus A/Puerto Rico/8/34 (PR8-OVA^29^; kindly provided by Dr S. Turner, University of Melbourne) in 30 μl PBS.

### Flow cytometry

Isolation of cells from lungs, MLNs and spleens, and surface staining was performed as described previously^54^. Cell suspensions from lungs were obtained by digestion with 2 mg ml^−1^ collagenase IV (Sigma-Aldrich) for 20 min in an air incubator at 37 °C. To obtain single-cell suspensions, tissue was passed through a metal cell strainer (80 μm; Sefer filters). Cells were washed with fluorescence-activated cell sorting (FACS) buffer (2% FCS, 2 mM EDTA and 0.02% sodium azide/1 × PBS) and incubated with anti-CD16/32 (2.4G2; BD Biosciences) for blocking Fc receptors. Cells were stained with biotin or fluorochrome-conjugated primary antibodies for 30 min on ice. Antibodies used for flow cytometry were purchased from BD Biosciences (CD8; 53-6.7, CD27; LG.7F9, CD44; IM7, CD45.2; 104), Biolegend (CXCR3; CXCR3-173, Ly6C; HK1.4), eBioscience (CD45.1; A20, CD62L; MEL-14, CD71; R17217, KLRG-1; 2F1, IL-7Rα; P84, IL-15Rα; DNT15Ra) or Invitrogen (SA-Alexa Fluor 594, SA-Alexa Fluor 647) and were used at a dilution of 1/400–1/2,000. After staining, cell suspensions were resuspended in 0.25 μg ml^−1^ propidium iodide (PI; Molecular probes) or 0.5 μg ml^−1^ 4′,6-diamidino-2-phenylindole (DAPI; Molecular probes) containing FACS buffer for exclusion of dead cells. Data were collected on a LSRII or Fortessa (BD Biosciences) and analysed with FlowJo software (Tree Star). Cell sorting was performed using a BD Aria II (BD Biosciences).

### BrdU labelling *in vivo*

On day 7 p.i., influenza virus-infected mice were intraperitoneally injected with 100 μl of 1 mg ml^−1^ BrdU (BD Biosciences) at 3, 5 and 8 h prior to tissue harvest. Cell suspensions from spleens of infected mice were stained for surface markers, and BrdU^+^ cells were identified using the BrdU Flow Kit according to the manufacturer’s specifications (BD Biosciences).

### Whole-mount confocal microscopy

On day 7 after infection, lungs were harvested and fixed in 10% sucrose, 4% formaldehyde solution at 4 °C overnight. The lungs were embedded in 4% agarose (Sigma-Aldrich) prepared in triple-distilled water and cut as 200-μm sections with a vibratome (Vibratome 1000 Classic; SDR). Staining was performed by incubating sections with purified anti-mouse CD45.2 antibody (eBioscience) and anti-laminin antibody (L9393; Sigma-Aldrich) overnight at 4 °C. The section was washed with 10% of FCS and incubated with Alexa Fluor 594 goat anti-rabbit IgG (Invitrogen) and Alexa Fluor 647 goat anti-rat IgG (Invitrogen) at 4 °C for 4 h. After washing, the sections were mounted on slides using Mounting medium (DAKO) and imaged with a SP5 confocal microscope (Leica Microsystems) with × 63 oil immersion objective (HCX PL APO 63 × /1.4−0.60 OIL Lbd Bl; Leica Microsystems). The maximum intensity z-projection images were generated using Volocity software (PerkinElmer).

### *In vitro* CD8^+^ T-cell stimulation and live-cell imaging

CD8^+^ T cells from spleen of naive Fucci/OT-I mice were purified and labelled with CTV as described above. Cells were stimulated with plate-bound anti-mouse CD3ε mAb (1.0 μg ml^−1^; 145-2C11; BD Biosciences) and anti-CD28 mAb (0.5 μg ml^−1^; 37.51; BD Biosciences) in T-cell medium (TCM) consisting of RPMI 1640, 10% of FCS, 1 mM sodium pyruvate, 10 mM HEPES, 100 U ml^−1^ penicillin, 100 μg ml^−1^ streptomycin and 50 μM 2-mercaptoethanol (Gibco). On the next day, cells were washed and cultured in TCM with 10 ng ml^−1^ rIL-2 (R&D Systems). The cells were then collected on day 2 or 4 after stimulation and stained with anti-CD8α-APC-Cy7 antibody and PI for dead-cell exclusion and sorted with a BD Aria II (BD Biosciences). PI-negative cells were further gated for CD8 positivity and into the 1st, 3rd and 8th generations according to the CTV dilution profile. Sorted cells were washed and resuspended in 10 ng ml^−1^ rIL-2 containing TCM and loaded onto microgrid arrays^55^ (Microsurfaces Pty. Ltd.) inside a four-well chamber coverglass slide (Lab-Tek II 155382; Nunc). The chamber slide was filled with 1,000 μl of rIL-2 containing (10 ng ml^−1^) TCM and set to an environment-controlled (37 °C, 5% CO_2_) TCS SP5 confocal microscope (Leica Microsystems). Time-lapse images were captured by 20 × 0.5 numerical apperture objective (HCX PL × 20/0.50 FLUOTAR; Leica) every 3–4 min for 66–90 h with 6–8 stacks at 0.8–1.2 μm of the z-plane.

### Single-cell tracking and analysis of time-lapse imaging

Time-lapse image files were converted into movie format using Volocity software (PerkinElmer) to track cells. Only wells containing single cells at the beginning of imaging were selected and tracked manually for sequential cell division until the end of imaging. If cells exited from the well or if other cells entered the same well, or if the cells died during imaging, wells were excluded from further analysis. Cell death was determined by cell morphology in bright-field images. The cell cycle times of tracked cells were measured and subjected to frequency distribution and correlation analysis using Spearman’s rank correlation coefficient^45^,^46^. For the permutation test, the raw data of cell cycle time from tracking cells in generation 8 were randomized and subjected to correlation analysis.

### *In vitro* stimulation with OVA peptide

Total splenocytes from Fucci/OT-I were incubated with K^b^-restricted ovalbumin-derived SIINFEKL peptide (100 ng ml^−1^, Auspep) in TCM for indicated times before FACS analysis.

### Quantitative real-time RT–PCR

Sorted populations from influenza virus-infected mice spleens were applied for quantitative PCR. Total RNA was extracted using Trizol reagent (Invitrogen) according to the manufacturer’s instructions. Reverse transcription (RT)–PCR was carried out with the Maloney MLV reverse transcriptase with Oligo (dT)_15_ Primer (Promega). Quantitative real-time PCR was performed using a Mx3000P qPCR system with MxPro qPCR software v2.0 (Stratagene) with SYBR Green JumpStart Taq ReadyMix (Sigma-Aldrich). The target specific primers were as follows: *mHPRT*, 5′-GTTGGAGACAGGCCAGACTTTGTTG-3′ and 5′-GAGGGTAGGCTGGCCTATAGGCT-3′; *mELF4*, 5′-CGGAAGTGCTTTCAGACTCC-3′ and 5′-GGTCAGTGACAGGTGAGGTA-3′; *mKLF2*, 5′-CCAACTGCGGCAAGACCTAC-3′ and 5′-AGTCGACCCAGGCTACATGTG-3′; *mS1P1R*, 5′-GTGTAGACCCAGAGTCCTGCG-3′ and 5′-AGCTTTTCCTTGGCTGGAGAG-3′; *mIFN-γ*, 5′-GAGGAACTGGCAAAAGGATG-3′ and 5′-TGAGCTCATTGAATGCTTGG-3′; *mIL-2*, 5′-GACACTTGTGCTCCTTGTCA-3′ and 5′-TCAATTCTGTGGCCTGCTTG-3′ (refs 56, 57).

### Whole-transcriptome microarrays

The larger or smaller sized cells of DAPI^−^CD8^+^ CD45.1^−^CD45.2^+^ cells from PR8-OVA infection on day 7 p.i. or *in vitro* culture after stimulation with SIINFEKL peptide (100 ng ml^−1^) were sorted with high purity. For CD8^+^ T-effector controls, cells were prepared from SIINFEKL-stimulated OT-I cells after 4 days *in vitro* culture. Effector memory (CD8^+^CD44^hi^CD62L^lo^) and central memory (CD8^+^CD44^hi^CD62L^hi^) cells were sorted from the spleens of influenza virus-infected C57BL/6 mice on day 42 p.i. Naive CD8^+^CD44^lo^CD62L^hi^ cells were sorted from uninfected C57BL/6 mice. Total RNA was extracted using the miRNeasy Micro Kit (Qiagen) from freshly sorted cells. The quality of RNA was checked using the RNA6000 Nano LabChip kit and 2100 Bioanalyzer (Agilent Technologies). RNA quantities were determined using the NanoDrop ND-1000 spectrophotometer (Thermo Scientific). Labelling, hybridization to Affymetrix GeneChip HT MG-430 PM Array Plate (Affymetrix) was performed by the Ramaciotti Centre for Gene Function Analysis (University of New South Wales, Sydney, Australia). Analysis of microarray data was performed using limma under the Bioconductor package in R^58^. Normalization was performed using the justRMA algorithm, and differential expression was calculated using the lmfit and ebayes models. The microarray data have been deposited to the National Center for Biotechnology Information Gene Expression Omnibus under accession number GSE48219. Gene set enrichment analysis ( www.broadinstitute.org/gsea) was performed to determine whether the predefined gene sets are enriched in the T-cell samples of interest by setting significant *P* less than 0.05 and false discovery rate less than 0.25 in the online tool.

### Statistical analyses

Unless otherwise indicated, the Student’s *t*-test (unpaired), Mann–Whitney *U*-test, two-way analysis of variance and *χ*^2^-test were performed using Prism software (Graphpad). Significance was assumed if *P*<0.05.

## Author contributions

I.K. and W.W. conceived the study. I.K., J.Q. and S.-Y.T. performed experiments. C.J.W. and P.M. performed correlation and inheritance analysis about the cell cycle time data. W.R. performed heatmap and cluster analysis of microarray data. L.L.C. helped with the animal ethics protocol and provided intellectual input. M.T., A.S.-S., O.K. and A.M. were involved in the generation and characterization of Fucci transgenic mice and discussion. P.D.H. gave critical comments on analysis and the manuscript. W.W. coordinated the project and wrote the manuscript together with I.K.

## Additional information

**Accession codes:** Microarray expression data have been deposited to NCBI’s Gene Expression Omnibus with accession number GSE48219.

**How to cite this article**: Kinjyo, I. *et al*. Real-time tracking of cell cycle progression during CD8^+^ effector and memory T-cell differentiation. *Nat. Commun.* 6:6301 doi: 10.1038/ncomms7301 (2015).

## Supplementary Material

Supplementary Information Supplementary Figures 1-6, Supplementary Note 1 and Supplementary Reference.

Supplementary Movie 1 Time-lapse imaging of individual Fucci CD8^+^ T cells. Cell Trace Violet-labeled Fucci-CD8^+^ T cells were stimulated with plate-bound anti-CD3ε (1.0 μg/ml) and anti-CD28 mAb (0.5 μg/ml) and sorted to collect the cells in the 8^th^ division generation. Sorted cells were seeded into microgrid arrays in the presence of rIL-2 (10 ng/ml) and subjected to time-lapse imaging every 3-4 min with 6-8 stacks at 0.8-1.2 μm (z-plane). Representative movie from 1^st^ day of imaging showing one area from multiple positioning. Movie duration: 16 h, Objectives: 20× 0.5 NA.

Supplementary Movie 2 Sequential divisions of smaller or larger sized T cells. Cell Trace Violet-labeled Fucci-CD8^+^ T cells were stimulated with plate-bound anti-CD3ε (1.0 μg/ml) and anti-CD28 mAb (0.5 μg/ml) and sorted to collect the cells in the 8^th^ division generation. Sorted cells were seeded into microgrid arrays in the presence of rIL-2 (10 ng/ml) and subjected to time-lapse imaging every 3-4 min with 6-8 stacks at 0.8-1.2 μm (z-plane). Representative movie with smaller and larger size cells from 3^rd^ day of imaging of sorted 8^th^ generation. Movie duration: 3.5 hr, Objectives: 20× 0.5 NA.
