# Supplementary material for: Real-time tracking of cell cycle progression during CD8+ effector and memory T-cell differentiation
Source: Nat Commun. 2015 Feb 24;6:6301. doi: 10.1038/ncomms7301 (PMC4346633; doi:10.1038/ncomms7301)
Supplement: Supplementary Information — Supplementary Figures 1-6, Supplementary Note 1 and Supplementary Reference. [file ncomms7301-s1.pdf]

SUPPLEMENTARY FIGURES

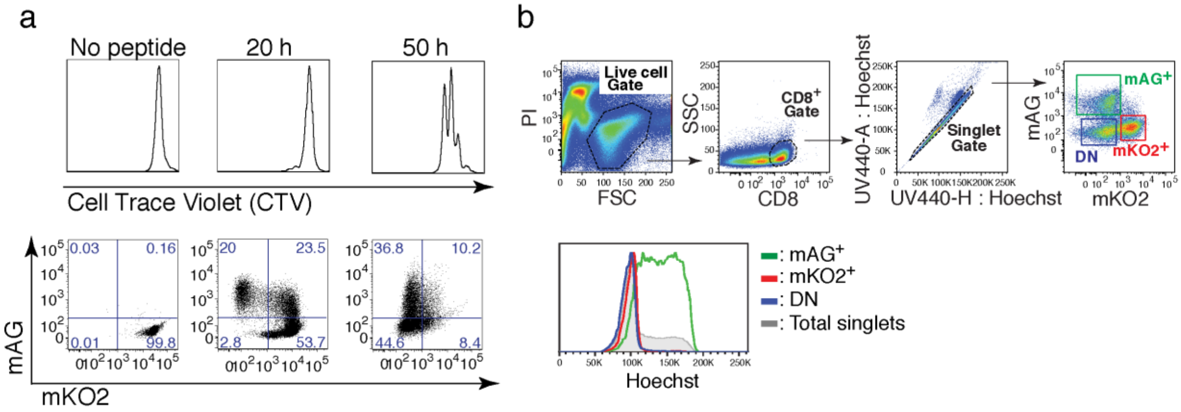

Supplementary Figure 1 | Fucci probes are sensitive indicators for cell cycle entry of OVA$_{257\text{-}264}$ SIINFEKL peptide stimulated Fucci/OT-I cells

(**a**) Flow cytometric analysis of mAG vs mKO2 expression in OVA$_{257\text{-}264}$ SIINFEKL peptide (100 ng/ml) stimulated Fucci/OT-I cells at the indicated time points. Total splenocytes of Fucci/OT-I mice were labeled with Cell Trace Violet (CTV) dye and stimulated *in vitro*. Data are representative of four independent experiments.

(**b**) Flow cytometric analysis of DNA profiling by Hoechst staining against mAG$^+$ or mKO2$^+$ or DN gated cells in Fucci CD8$^+$ T cells at 40 h after *in vitro* stimulation [plate-bound anti-CD3ε (1.0 μg/ml) and anti-CD28 mAb (0.5 μg/ml)]. Data are representative of three independent experiments.

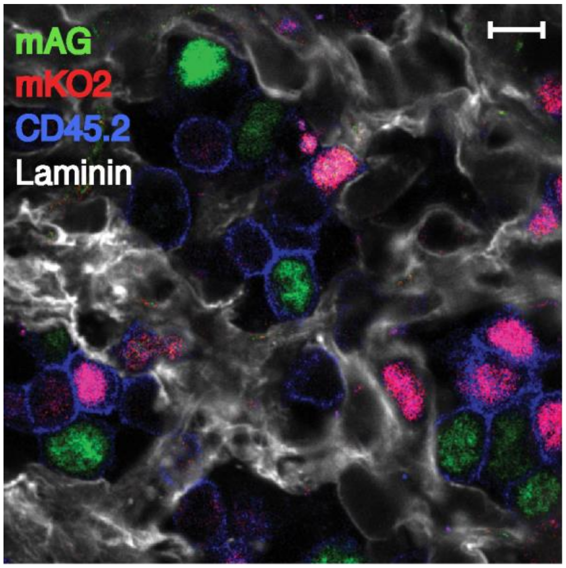

Supplementary Figure 2 | The presence of mAG$^+$ Fucci/OT-I cells in the lungs on day 7 p.i. reveals cycling virus-specific CD8$^+$ T cells at the effector sites

Magnetic-Activated Cell Sorting (MACS) purified Fucci/OT-I (CD45.2$^+$) cells were transferred to CD45.1$^+$ recipients prior to intranasal (i.n.) PR8-OVA influenza A virus infection. Vibratome sections of the lungs day 7 p.i. were stained with anti-CD45.2 (blue) and anti-laminin (white) antibodies and imaged with a confocal microscope using 63× oil immersion 1.4 NA objective (Scale bar; 7.0 μm). The image is representative of three independent experiments.

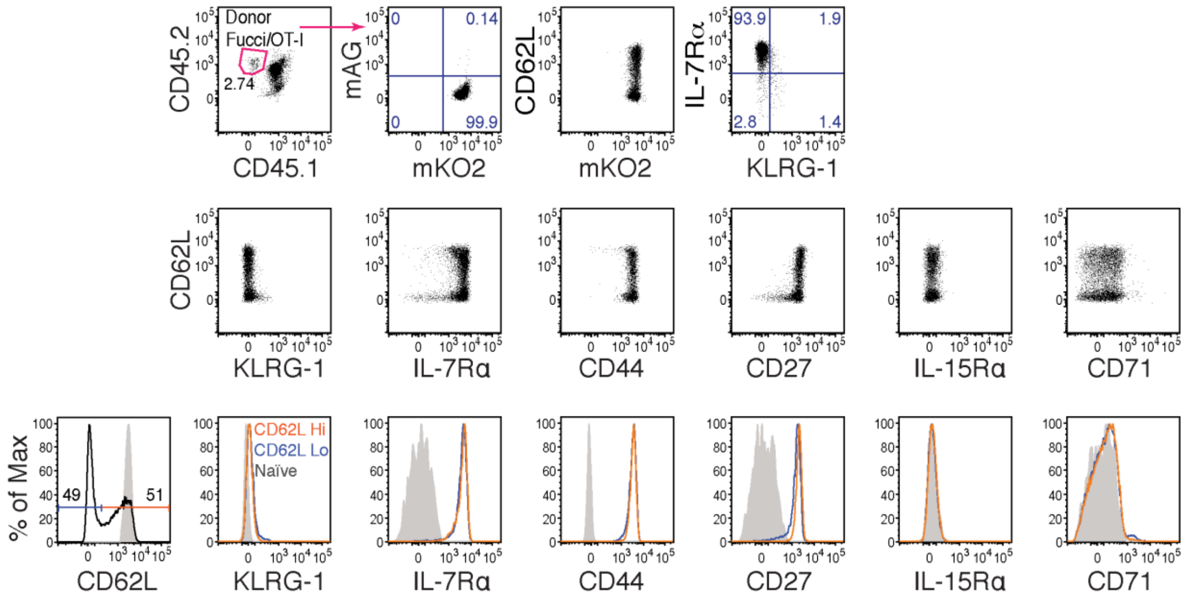

Supplementary Figure 3 | Comparison of memory markers in CD62L^{hi} and CD62L^{lo} cells on day 32 p.i.

MACS purified Fucci/OT-I (CD45.2^{+}) cells were labeled with CTV dye and transferred to CD45.1^{+} recipients prior to PR8-OVA influenza A virus infection. Flow cytometric analysis of memory T cell marker on transferred Fucci/OT-I cells in spleens on day 32 p.i. of PR8-OVA infection. Top two rows: Representative plots of the expression of mAG vs mKO2, IL-7Rα vs KLRG-1, and CD62L vs other memory markers. Bottom row: The expression level of each marker was compared between CD62L^{hi} and CD62L^{lo} gated population. Data are representative of two independent experiments with three mice.

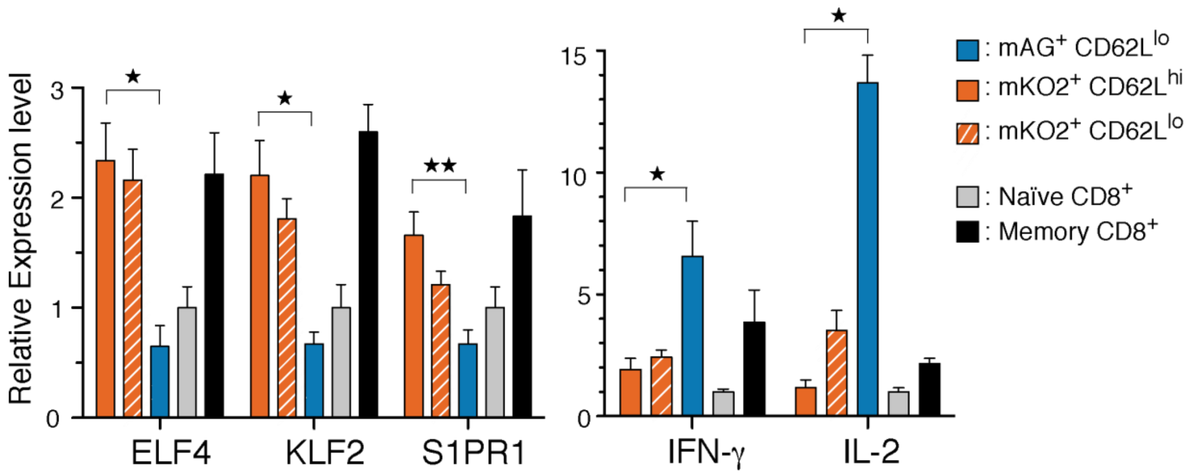

Supplementary Figure 4 | Expression levels of memory or effector T cell related genes in subsets of virus specific CD8^{+} T cells on day 7 p.i.

MACS purified Fucci/OT-I (CD45.2^{+}) cells were transferred to CD45.1^{+} recipients prior to PR8-OVA influenza A virus infection. mAG^{+}CD62L^{lo}, mKO2^{+}CD62L^{lo} or mKO2^{+}CD62L^{hi} CD8^{+} T cells were sorted from spleens on day 7 p.i. For control samples, naïve (CD44^{lo}CD122^{lo}) and memory (CD44^{hi}CD122^{hi}) CD8^{+} T cells were sorted from naïve C57BL/6 wild-type mice. Values represent the mean ± S.E.M. of triplicate determinations normalized to the expression level of hypoxanthine-guanine phosphoribosyltransferase. Data are summarized from three independent sorted samples from three independent experiments. ★$P < 0.001$ versus mAG^{+} CD62L^{lo} (Student's *t*-test).

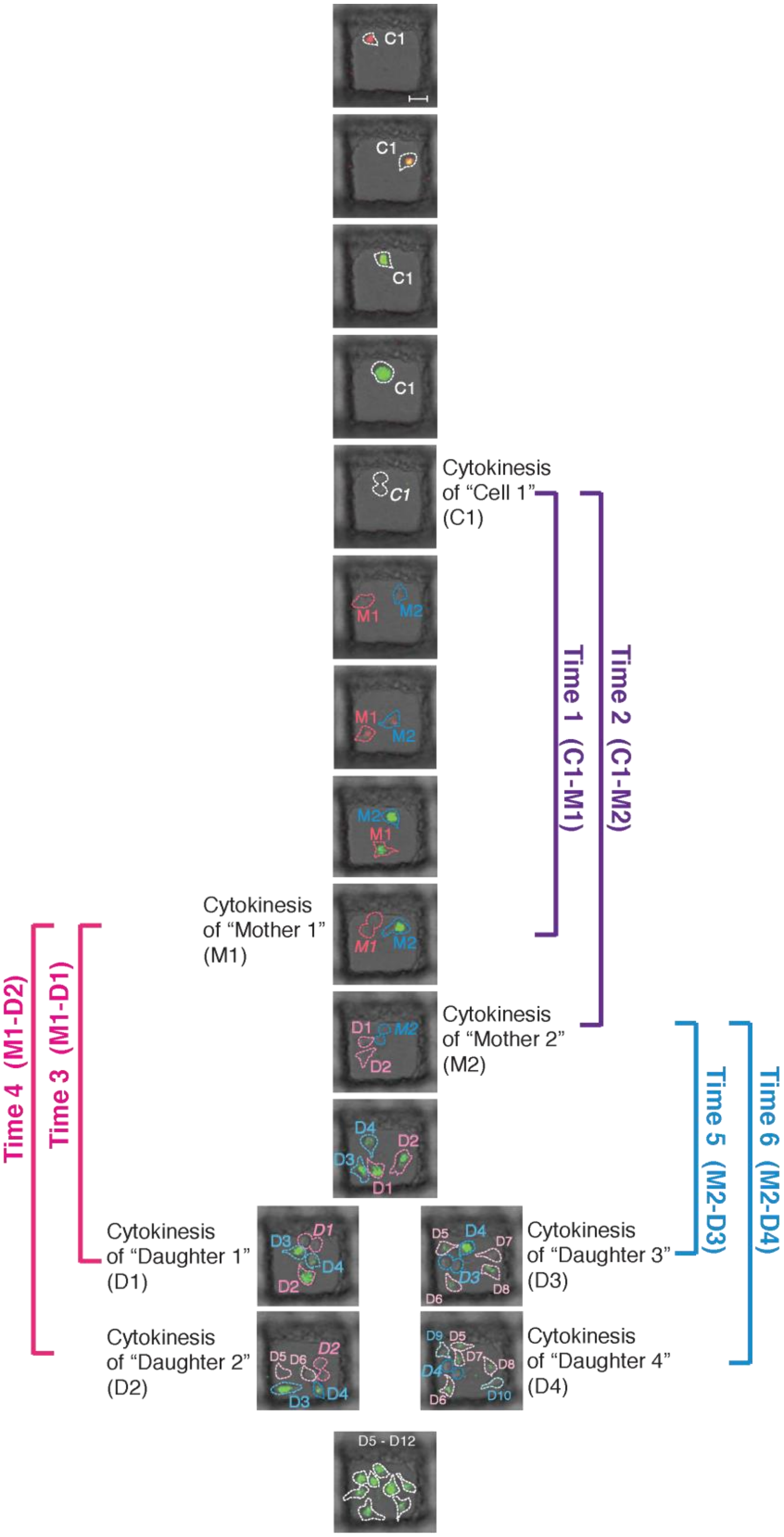
C1
Cytokinesis of "Cell 1" (C1)
M1
M2
Time 1 (C1-M1)
Time 2 (C1-M2)
Cytokinesis of "Mother 1" (M1)
Cytokinesis of "Mother 2" (M2)
D1
D2
D3
D4
Time 3 (M1-D1)
Time 4 (M1-D2)
Time 5 (M2-D3)
Time 6 (M2-D4)
Cytokinesis of "Daughter 1" (D1)
Cytokinesis of "Daughter 2" (D2)
Cytokinesis of "Daughter 3" (D3)
Cytokinesis of "Daughter 4" (D4)
D5
D6
D7
D8
D9
D10
D5 - D12

Supplementary Figure 5 | Scheme of time-lapse imaging for tracking sequential cytokinesis from single cells

The initial cell (C1) divided into two mother cells (M1 and M2), which were tracked for next cytokinesis of each daughter cell (D1, D2, D3 and D4) and individual cell cycle times were measured (Time 1 = C1−M1, Time 2 = C1−M2 and Time 3 = M1−D1, Time 4 = M1−D2, Time 5 = M2−D3, Time 6 = M2−D4) for further analysis. The snapshots are shown from representative movie (**Supplementary Movie 1**) from three independent experiments are shown (Scale bar; 10.0 μm).

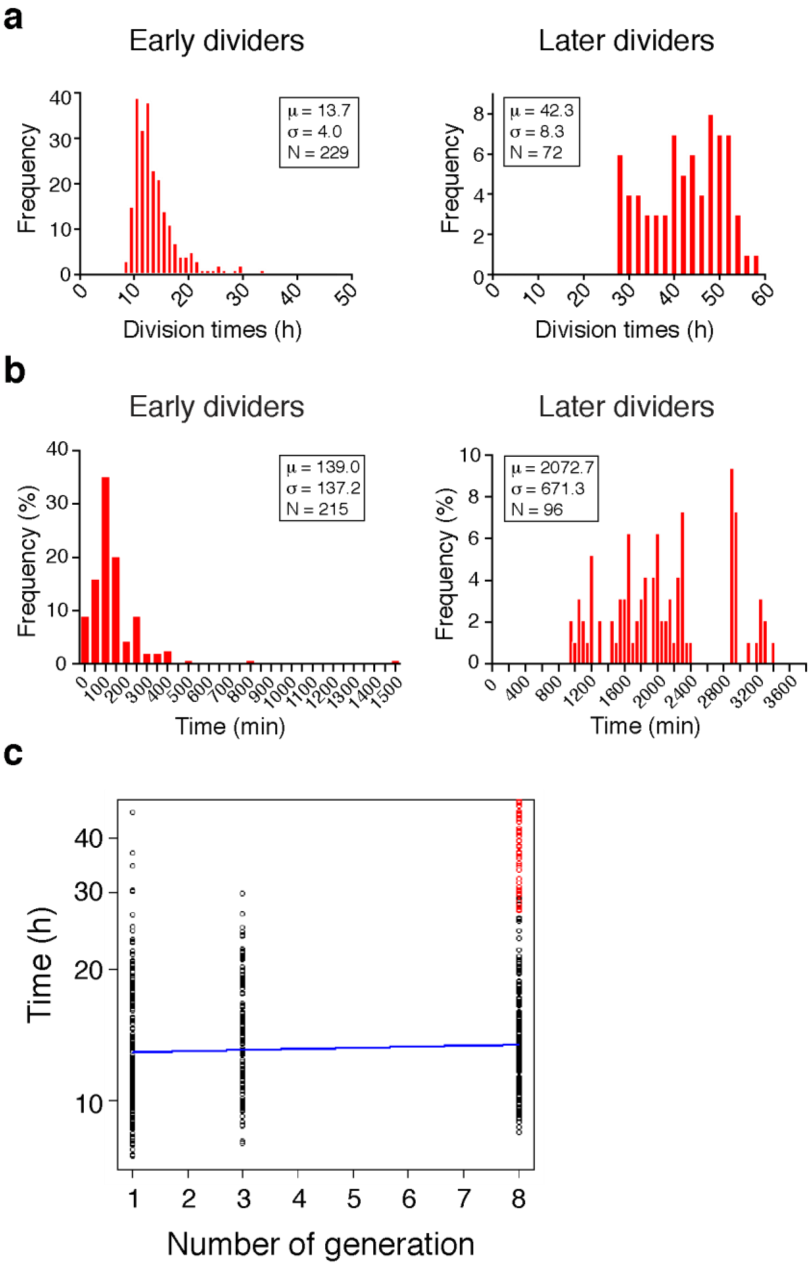

Supplementary Figure 6 | Two distinctive cell cycle time shown by the cells from the 8th generation

(**a**) The frequency of total cell cycle time of "early dividers" (divided on 1st day of imaging)

and “later dividers” (divided on 2nd day of imaging) in the 8th generation. The data of “early dividers” (Left panel) and “later dividers” (Right panel) are shown in each histogram with mean (μ), standard deviation (σ) and number of events (N). Data were summarized from three independent experiments from **Figure 4**.

(**b**) The percentage of frequency distribution for $mKO2^+$ phase duration of cells from the 8th generation. The data of divided cells found on 1st imaging day (Early dividers; Left panel) and ones found on 2nd imaging day (Later dividers; Right panel) are shown in each histogram with mean (μ), standard deviation (σ) and number of events (N). Data were summarized from three independent experiments from **Figure 4**.

(**c**) A linear model of log-transformed division time against cell generation number for early dividers provides no significant evidence for a systematic increase in division time with cell generation (p = 0.15). A two-sample t-test was performed on the log-transformed division times of early and later dividers from the 8th generation as well as the 1st and 3rd generations. The result rejected the null hypothesis, i,e, that there is no difference in the mean values of the two distribution, with a p-value of $p < 2.2\times10^{-16}$.

SUPPLEMENTARY NOTES

Supplementary Note 1: The double positive (DP) and double negative (DN) phase in the Fucci system

The Fluorescent Ubiquitination-based Cell Cycle Indicator (Fucci) was originally developed to overcome limitations for marking the G_1-S phase transition in living cells (Fig. 1a). By exploiting the reciprocal degradation of Cdt1 and Geminin based on ubiquitination in G_1 or $S/G_2/M$ phase, respectively, Fucci probes mKO2-hCdt1(30/120) and mAG-hGeminin(1/110) were employed for transgenic mice to longitudinally track the cell cycle status of cells of interest *in vivo* and *ex vivo*. As reported[26], mKO2-hCdt1(30/120) expression specifies G_0/G_1 phase and is degraded after cell entry into S phase. Oppositely, mAG-hGeminin(1/110) accumulates through $S/G_2/M$ phases. Based on this reciprocal regulation, G_1 cells can be determined by mKO2 fluorescent positivity and cells in $S/G_2/M$ phases by mAG positivity. The quiescent G_0 phase cells are accompanied by a high accumulation of mKO2-hCdt1(30/120), which is reflected by the high intensity of mKO2[28]. In the current study, we also found that $CD8^+$ T cells from Fucci transgenic mice showed higher intensity of mKO2 when they are in G_0 phase than G_1 phase of fast cycling cells. $CD8^+$ T cells in the memory phase reverse to a bright $mKO2^+$ status consistent with the high accumulation of mKO2-hCdt1(30/120) during quiescent G_0 phase.

With degradation of mKO2-hCdt1(30/120) and concomitant increase of mAG-hGeminin(1/110) at the onset of S phase, cells in the early S phase become double-positive (DP). After degradation of mAG-hGeminin(1/110) at late M phase and until mKO2-hCdt1(30/120) accumulates in G_1 phase up to the detectable level, cells are remain double negative (DN). The induction of Fucci proteins is constant under the constitutively active promoter, so the amount of Fucci is mostly regulated by ubiquitination-based degradation[26]. Based on this design, it is conceivable that the intensity of Fucci probes (mKO2-hCdt1(30/120) and mAG-hGeminin(1/110)) can be changed depending on the duration of G_1 and S/G_2M phases when Fucci transduced cells change cell cycle duration. It should also be noted that we cannot formally exclude the possibility that

DN states in the current transgenic mice may result from a certain degree of heterogeneity in Fucci transgene expression, in contrast to highly-clonal stable Fucci-expressing cell lines described previously[28,59].

SUPPLEMENTARY REFERENCE

59. Abe, T. *et al*. Visualization of cell cycle in mouse embryos with Fucci2 reporter directed by Rosa26 promoter. *Development*, **140**, 237-246 (2013).
